# Intestinal permeability in human cardiovascular diseases: a systematic review and meta-analysis

**DOI:** 10.3389/fnut.2024.1361126

**Published:** 2024-07-17

**Authors:** Jiang-Hong Xiao, Yu Wang, Xi-Mei Zhang, Wen-Xiao Wang, Qiao Zhang, Yu-Ping Tang, Shi-Jun Yue

**Affiliations:** ^1^Key Laboratory of Shaanxi Administration of Traditional Chinese Medicine for TCM Compatibility, Shaanxi University of Chinese Medicine, Xi’an, China; ^2^International Joint Research Center on Resource Utilization and Quality Evaluation of Traditional Chinese Medicine of Hebei Province, Hebei University of Chinese Medicine, Shijiazhuang, China

**Keywords:** cardiovascular diseases, intestinal permeability, meta-analysis, LPS, zonulin

## Abstract

**Background:**

There is a link between cardiovascular diseases and intestinal permeability, but it is not clear. This review aimed to elucidate intestinal permeability in cardiovascular diseases by meta-analysis.

**Methods:**

Multidisciplinary electronic databases were searched from the database creation to April 2023. All included studies were assessed for risk of bias according to the Joanna Briggs Institute Critical Appraisal Checklist. The heterogeneity of each study was estimated using the I^2^ statistic, and the data were analyzed using Review Manager 5.3 and Stata 16.0.

**Results:**

In total, studies in 13 pieces of literature were included in the quantitative meta-analysis. These studies were conducted among 1,321 subjects mostly older than 48. Patients had higher levels of intestinal permeability markers (lipopolysaccharide, d-lactate, zonulin, serum diamine oxidase, lipopolysaccharide-binding protein, intestinal fatty acid binding protein, and melibiose/rhamnose) than controls (standard mean difference SMD = 1.50; 95% CI = 1.31–1.88; *p* < 0.00001). Similarly, lipopolysaccharide levels were higher in patients than in controls (SMD = 1.61; 95% CI = 1.02–2.21; *p* < 0.00001); d-lactate levels were higher in patients than in controls (SMD = 1.16; 95% CI = 0.23–2.08; *p* = 0.01); zonulin levels were higher in patients than in controls (SMD = 1.74; 95% CI = 1.45–2.03; *p* < 0.00001); serum diamine oxidase levels were higher in patients than in controls (SMD = 2.51; 95% CI = 0.29–4.73; *p* = 0.03).

**Conclusion:**

The results of the meta-analysis verified that the intestinal barrier was damaged and intestinal permeability was increased in patients with cardiovascular diseases. These markers may become a means of the diagnosis and treatment of cardiovascular diseases.

**Systematic review registration:**

https://www.crd.york.ac.uk/PROSPERO/display_record.php?RecordID=414296, identifier CRD42023414296.

## 1 Introduction

Cardiovascular diseases (CVDs) are widespread in older people and are becoming younger with an increasing incidence worldwide (1, 2). CVDs are characterized by systemic vascular lesions and vascular lesions concentrated in the brain and heart. The incidence of CVDs is affected by many factors, such as genetics, living habits, diet, etc. In recent years, more and more pieces of literature have shown that intestinal microbiota and barrier dysfunction are involved in the development and progression of CVDs. Although the researchers pay more attention to improving the outcome of CVDs by controlling microbiota (3), the common risk factors of CVDs, such as diabetes (4), obesity (5), nonalcoholic fatty liver disease (6), and hyperlipidemia (6), have been reported to damage intestinal barrier and increase intestinal permeability (IP). Therefore, the interaction between IP and CVDs should also be given full attention.

The intestinal barrier is a complex multi-layer structure, including the luminal mucus layer, the intestinal epithelial layer, and the inner layer of the mucosal immune system (5). The intestinal epithelial barrier is composed of monolayer intestinal epithelial cell junctions. Tight junctions and their related proteins, including occlusive zone, occluding, and claudin, are important factors in the formation of the intestinal epithelial barrier (7, 8). The integrity of the epithelial barrier is crucial to protect the host from immune inflammation and the invasion of harmful microbiota and metabolites. Once intestinal epithelial cells die or apoptosis, the tight junction is destroyed or mucus degradation is maladjusted, which will lead to an increase in IP (8). Besides, trauma, infection, ischemia, and reperfusion injury can also lead to the increased IP. The increase in IP, however, can promote the translocation of harmful substances and pathogens into the blood, thus enhancing the systemic inflammation response (9).

To evaluate IP, both the *in vitro* and *in vivo* methods have been used. The *in vitro* methods include chamber technique, measurement of transepithelial electrical resistance, and so on. The *in vivo* evaluation methods include measuring urine excretion after oral administration of the probe, observation of gap between epithelial cells by confocal laser micro endoscopy after the application of a fluorescent agent, and detection of blood biomarkers of IP (10, 11). Till now, blood biomarkers such as lipopolysaccharide (LPS), LPS-binding protein (LBP), zonulin, diamine oxidase (DAO), intestinal fatty acid binding protein (I-FABP), citrulline, d-lactate, are often used clinically (3, 9). For example, oral probes or blood biomarkers have been widely used to reflect IP in diseases such as psychiatric disorders, gastrointestinal disorders, and nonalcoholic fatty liver disease (10–12).

Research on IP for cardiometabolic diseases has just begun, and the results of animal and human studies tend to increase IP in cardiovascular disease, but it is not clear enough. At present, there is a lack of randomized controlled trials on CVDs and IP, so this review summarized the cross-sectional study and discusses whether IP increases in patients with CVDs. For the first time, this study used meta-analysis to link CVDs to IP, to get a more integrated conclusion. We also discussed the relationship between the roles of IP, gut microbiota, and CVDs, as well as other factors that influence IP. The results of our analysis could provide new ideas for the treatment of CVDs and hopefully raise the importance of IP to potential researchers.

## 2 Methods

The systematic review and meta-analysis details of this cross-sectional study are in the International Registry of Prospective Systematic Reviews (PROSPERO; No. CRD42023414296). This systematic review follows the PRISMA guidelines.

### 2.1 Search strategy

The literature search was performed in these databases: PubMed, Embase, Cochrane Library, Web of Science, ClinicalTrials.gov, Wanfang, Weipu, China National Knowledge Infrastructure, and China Biology Medicine disc. The search time was set from the database creation to April 2023. The keywords and Medical Subject Headings (MeSH) terms searched included: coronary heart disease, CVDs, atherosclerosis, myocardial infarction, hypertension, IP, and intestinal barrier function. Then, the Boolean operators AND and OR are used to combine the search words (refer to Supplementary material).

### 2.2 Inclusion and exclusion criteria

Inclusion criteria:

- P (Population): general population.
- I (Exposure/Intervention): diagnosis of CVDs.
- C (Comparison): healthy subjects without CVDs.
- O (Outcome): the outcome index was the level of LPS, d-lactate, zonulin, DAO, LBP, I-FABP, and melibiose/rhamnose.
- S (Study Design): the cross-sectional study.
- Exclusion Criteria:
- Letters, conference abstracts, newsletters, meta-analyses, and review articles.
- Animal experiments.
- Dissertation of degree.
- There were no healthy controls.
- Unable to obtain data for outcome indicators.

### 2.3 Literature selection and data extraction

After using software and manually deleting duplicates, the literature was screened in the order of reading the title and abstract first and then reading the full text. The standard table was used to extract the contents of the included literature, including the name of the first author, year of publication, country, average age, sex ratio, type of disease, and outcome index. Means and standard deviations of outcome indicators were extracted and combined when multiple subgroups of CVDs were present using the formula. When the data were incomplete, researchers attempted to contact the corresponding authors to obtain the needed information. Sensitivity analysis was performed by sequentially deleting individual literature and rerunning the analysis. To reduce the selectivity bias, the above process was carried out independently by two researchers. When there was disagreement, the third researcher decided.

### 2.4 Assessment of quality

Two researchers evaluated the risk of bias using the Joanna Briggs Institute (JBI) manual, and when there was disagreement, the third researcher decided. The risk of bias instrument consisted of eight items for which the answer is “yes,” “no,” “unclear” or “not applicable.” If the answer was yes, the question was assigned a score of 1. If not, it was assigned a score of 0. Total quality scores ≥6, 4 to 5, and ≤ 3 were regarded as low, moderate, and high risk, respectively.

### 2.5 Statistical analysis

The software used in the analysis were Review Manager 5.3 and Stata 16.0. The outcome indicators involved in this study were all continuous variables. Due to the differences in the measurement units of the included studies, standardized mean difference (SMD) was used as the effect size. The pooled results were presented as SMDs and 95% confidence (95% CI) for each effect size. Heterogeneity was calculated using χ^2^ and I^2^. When the statistical heterogeneity of each study was small (*p* > 0.1, I^2^ < 50), the fixed effect model was used. Otherwise, a random effects model was used. If the mean and standard deviation are not provided in the included literature, Eq. (1) and (3) were used for conversion; if there are multiple subgroups in the study, Eq. (2) was used for merging (13, 14). The merged results were presented in the form of forest plots, with statistical significance when *p* < 0.05. The Funnel plot and Egger test were used to detect publication bias.


(1) $$SD=SE\times \sqrt{N}$$

(2) $$SD=\sqrt{\frac{\left(N_{1}-1\right)SD_{1}^{2}+\left(N_{2}-1\right)SD_{2}^{2}+\frac{N_{1}N_{2}}{N_{1}+N_{2}}\left(M_{1}^{2}+M_{2}^{2}-2M_{1}M_{2}\right)}{N_{1}+N_{2}-1}}$$

(3) When data were expressed using the median and interquartile range, we converted the data to mean and SD using the formula proposed by Wan et al. (15).

Where SD is the standard deviation, SE is the standard error, N is the sample size, and M is the average value.

## 3 Results

### 3.1 Selection process

**Figure 1:**
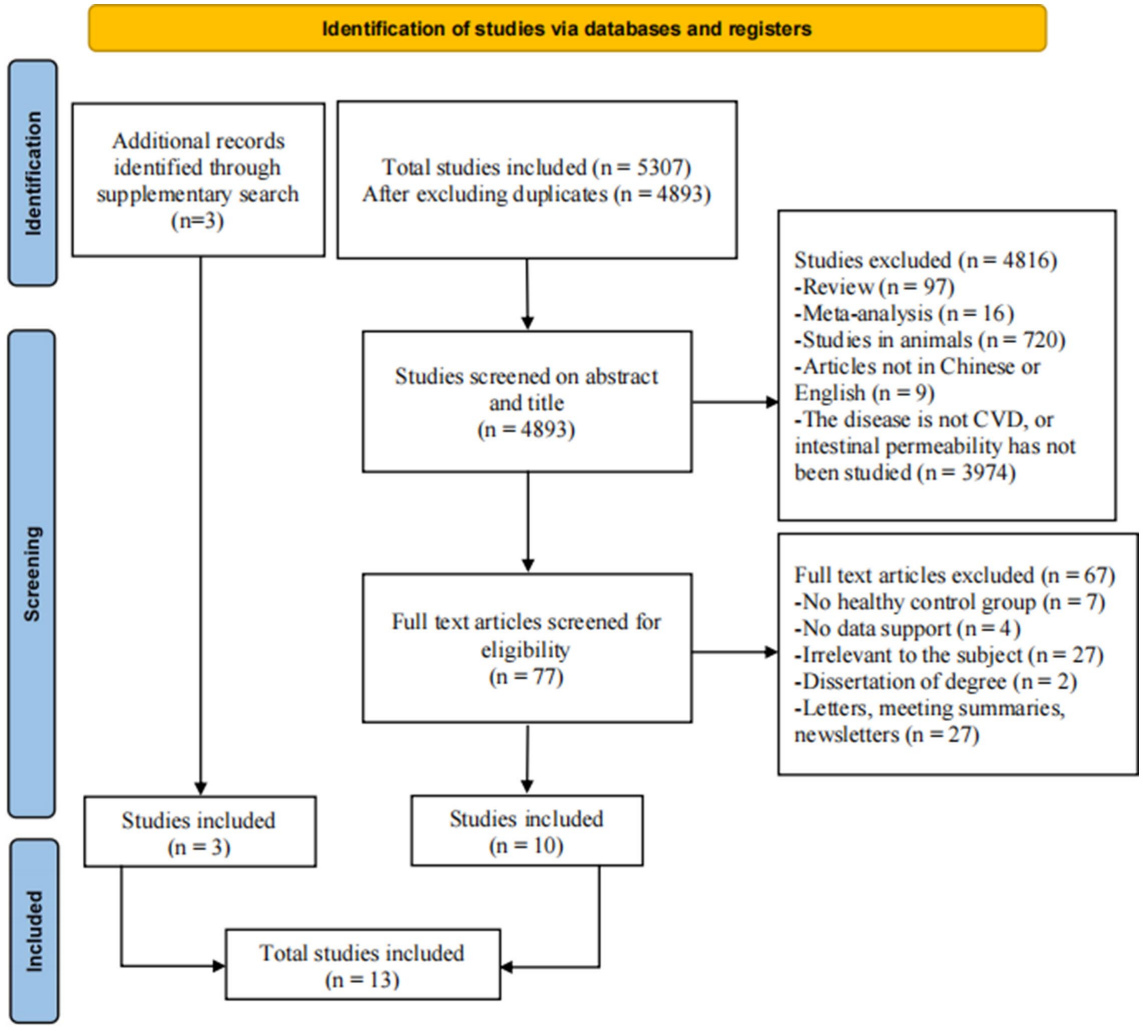
The PRISMA flow diagram of the study.

As shown in the PRISMA diagram (Figure 1), in total, 5,307 pieces of literature were retrieved from the database, and 414 duplicates were excluded by Endnote X9 software and manual. Among the remaining 4,893 pieces of literature, 4,816 pieces of literature were excluded by browsing their titles and abstracts. After browsing the full text, 67 pieces of literature were excluded. Finally, 10 pieces of literature met the requirements. Additional searches revealed three pieces of literature. In total, 13 pieces of literature were included.

### 3.2 Research characteristics

In total, 13 pieces of literature include 22 studies involving 815 patients with CVDs and 506 controls. Supplementary Table S1 summarized the characteristics of the included studies. The proportion of male subjects ranged from 25 to 76%. Each study’s sample size was significantly different, with a minimum of 25 subjects and a maximum of 206 subjects. The types of CVDs included in the study included seven types (acute type A aortic dissection, atherosclerosis, chronic heart failure, coronary artery disease, coronary heart disease, hypertension, and microvascular angina). The patients in the five pieces of literature had coronary heart disease, the patients in the three pieces of literature had chronic heart failure, and the diseases of the patients in the rest of the literature were different. The subjects in one of the studies were Americans (16), and the subjects in the four studies were Europeans (17–20), and the subjects in the eight studies were Asians (21–28). The reporting time of all the literature was from 1999 to 2023. All literature included IP, of which 10 pieces of literature took it as the main research content, two pieces of literature mainly studied intestinal microbiota (22, 25), a piece of literature mainly studied the inflammatory mechanism of atherosclerosis but IP was a secondary research content (17).

### 3.3 Quality of included studies and risk of bias

All items of the JBI Critical Appraisal Checklist applied to this study and the risk of bias identification of the included cross-sectional studies are shown in Supplementary Table S2. Four pieces of literature showed moderate risk and nine pieces of literature showed low risk.

### 3.4 Meta-analysis

**Figure 2:**
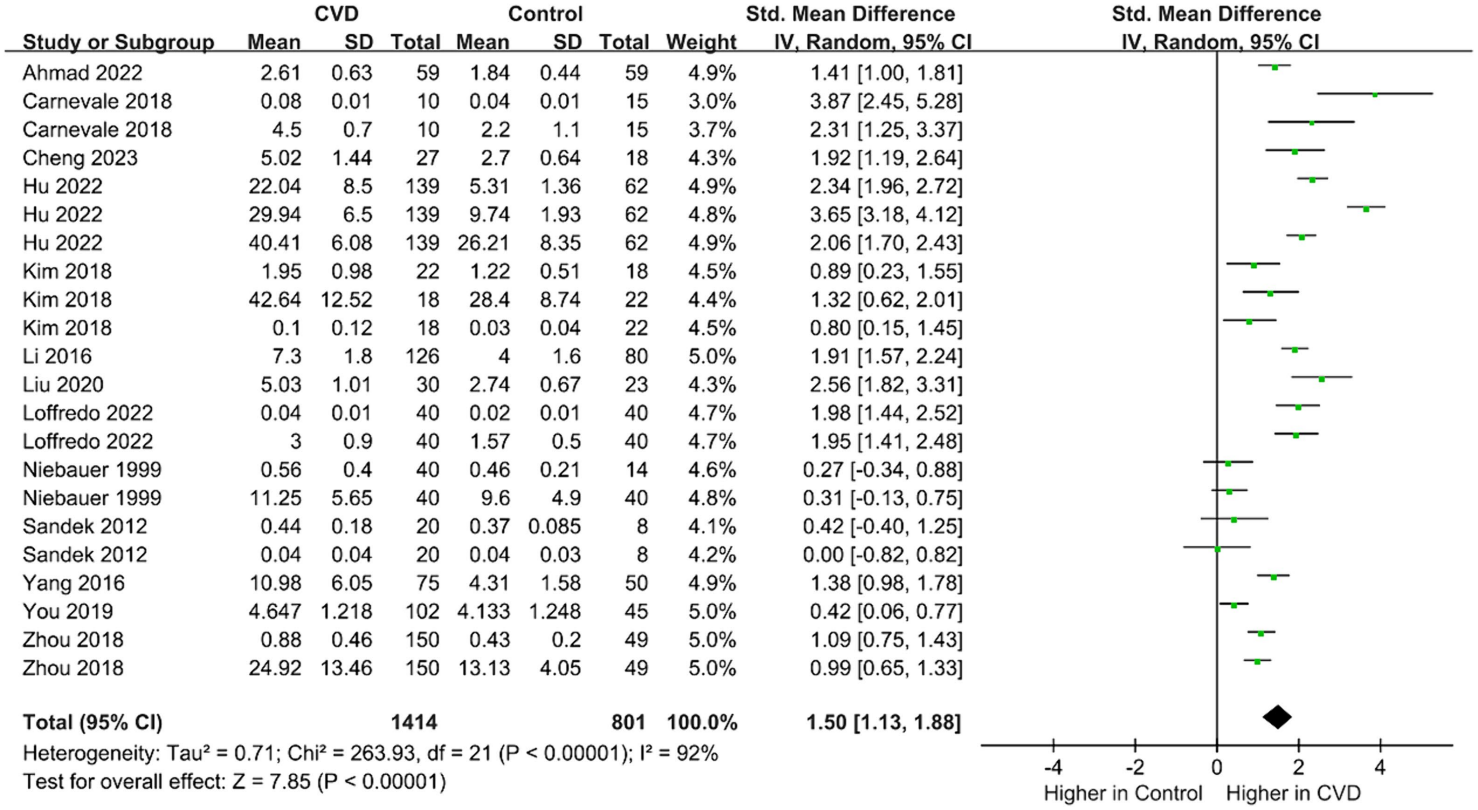
Forest plot for assessing IP marker levels in patients vs. controls.

The mean IP marker levels are quantitatively synthesized in Figure 2. The outcome indicators involved in this study included LPS, d-lactate, zonulin, serum DAO, LBP, I-FABP, and melibiose/rhamnose levels. Data on the levels of IP markers in patients (n = 815) and controls (n = 506) were provided in 13 studies. Overall, patients vs. controls had an increased IP using IP markers (SMD = 1.50; 95% CI = 1.31–1.88; *p* < 0.00001). Because the measurement methods of IP markers were different, the random effect model was used. In addition, to detect publication bias, a funnel plot and Egger test were depicted (Supplementary material). The funnel plot and Egger test demonstrated that there was no potential publication bias among studies.

### 3.5 Meta-analysis of the main markers

#### 3.5.1 LPS

**Figure 3:**
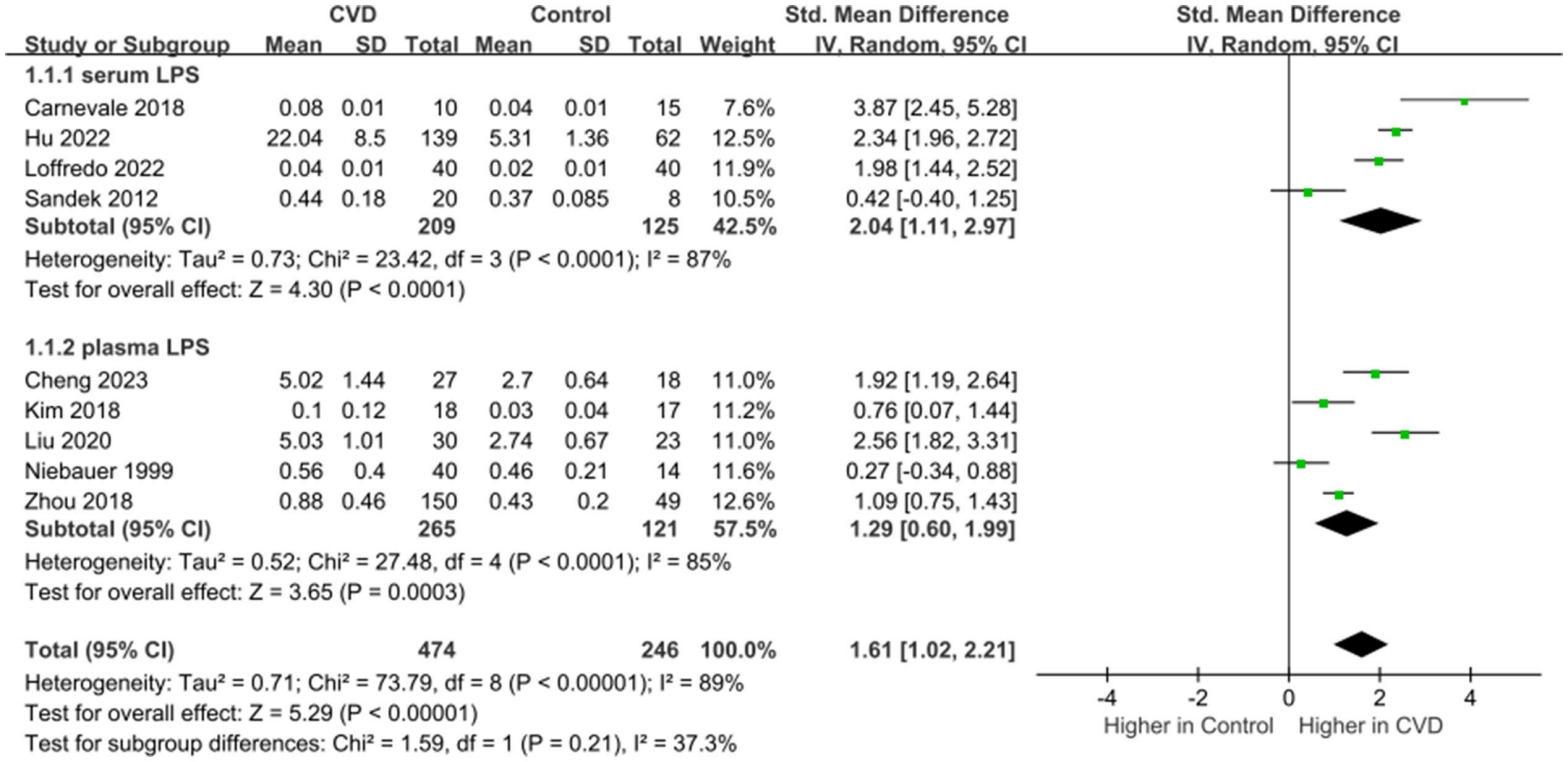
Forest plot for assessing LPS levels in patients vs. controls.

LPS in the outer membrane of gram-negative bacteria can activate and aggravate the inflammatory response after binding to related receptors (18). Inflammation induces intestinal barrier dysfunction, which in turn promotes the invasion of LPS. As a reliable indicator of both inflammation and IP, data on the levels of LPS in patients (n = 474) and controls (n = 246) were provided in nine studies (Figure 3) (16–20, 22, 23, 25, 28). Overall, LPS levels were higher in patients than in controls (SMD = 1.61; 95% CI = 1.02–2.21; *p* < 0.00001), with evidence of high heterogeneity (I^2^ = 89%). The subgroup analysis showed that patients and the controls had significantly different levels of serum (SMD = 2.04; 95% CI = 1.11–2.97; *p* < 0.0001) and plasma LPS (SMD = 1.29; 95% CI = 0.60–1.99; *p* = 0.0003), but there was a greater difference in serum LPS between the two groups. The sequential exclusion of the included studies did not reduce heterogeneity.

#### 3.5.2 D-lactate

**Figure 4:**
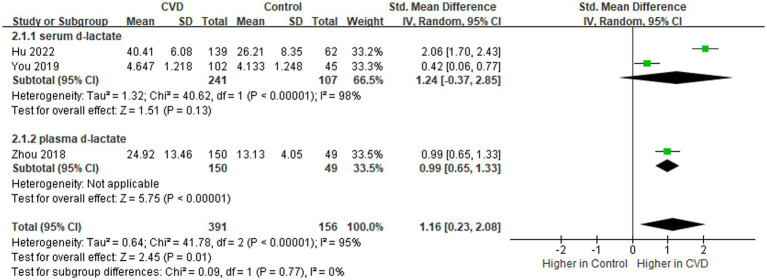
Forest plot for assessing d-lactate levels in patients vs. controls.

D-lactate is a metabolite of bacteria in the intestine, which could enter the circulation with the increase of IP (29) and is currently used as a marker for judging increased IP (28). Data on the levels of d-lactate in patients (*n* = 391) and controls (n = 156) were provided in three studies (Figure 4) (23, 27, 28). Overall, d-lactate levels were higher in patients than in controls (SMD = 1.16; 95% CI = 0.23–2.08; *p* = 0.01), with evidence of high heterogeneity (I^2^ = 95%). In the subgroup analysis, no significant difference in serum d-lactate levels in patients compared to controls (SMD = 1.24; 95% CI = −0.37-2.85; *p* = 0.13). The sequential exclusion of the included literature did not reduce heterogeneity.

#### 3.5.3 Zonulin

**Figure 5:**
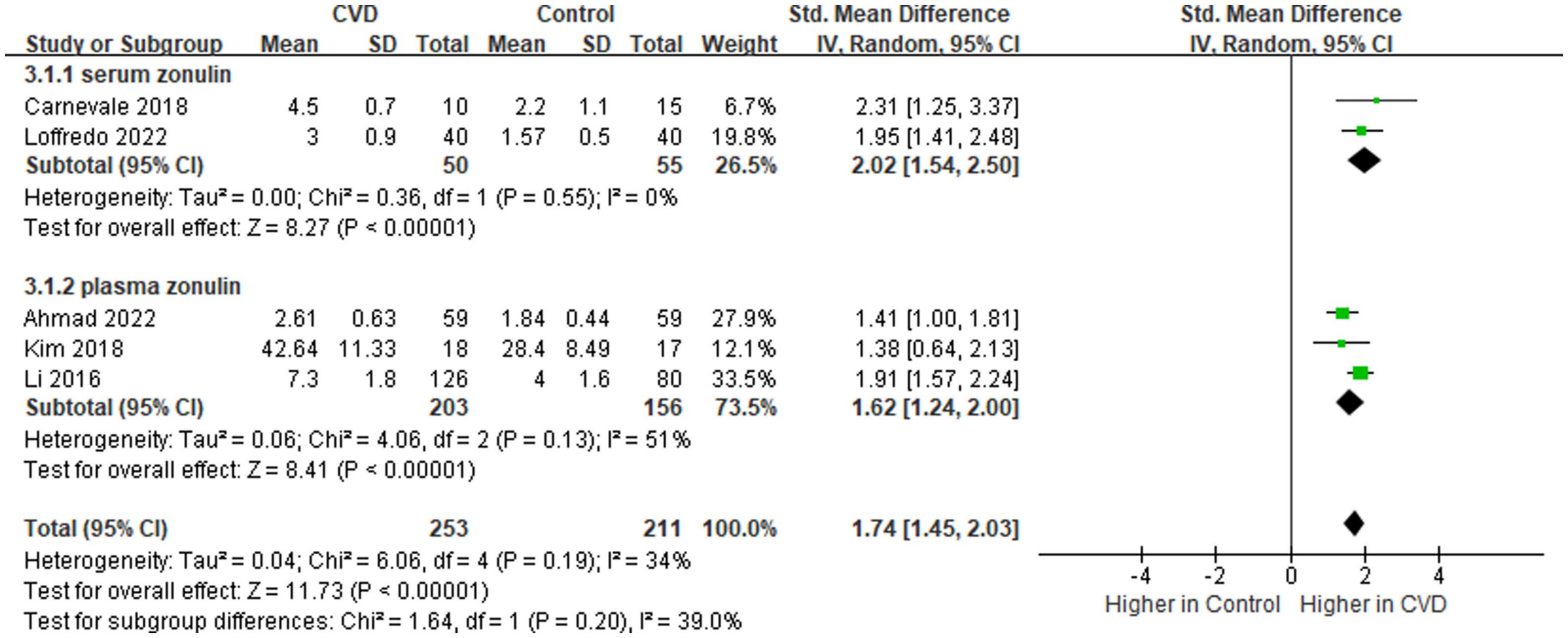
Forest plot for assessing zonulin levels in patients vs. controls.

Zonulin is the only physiological regulator that regulates IP by breaking down tight junctions between cells (30). Its upregulation leads to the destruction of barrier function, resulting in an uncontrolled inflow of diet and microbial antigens (30). Data on the levels of zonulin in patients (n = 253) and controls (n = 211) were provided in five studies (Figure 5) (16–18, 21, 24). Overall, zonulin levels were higher in patients than in controls (SMD = 1.74; 95% CI = 1.45–2.03; *p* < 0.00001), with evidence of low heterogeneity (I^2^ = 34%). In the subgroup analysis, both serum (SMD = 2.02; 95% CI = 1.54–2.50; *p* < 0.00001) and plasma zonulin levels (SMD = 1.62; 95% CI = 1.24–2.00; *p* < 0.00001) were observed to be increased in patients compared to controls.

#### 3.5.4 Serum DAO

**Figure 6:**
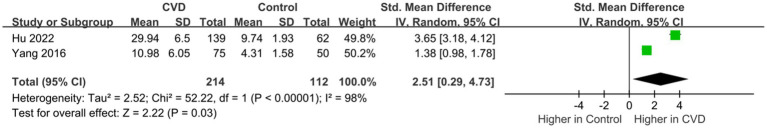
Forest plot for assessing serum DAO levels in patients vs. controls.

DAO is an intracellular enzyme in the mucosal villous epithelial cells. Following damage, necrosis, and exfoliation of the intestinal mucosal cells, DAO could translocate into blood, suggestive of destruction of the intestinal mucosal barrier and changes in IP (31). Data on the levels of serum DAO in patients (*n* = 214) and controls (*n* = 112) were provided in two pieces of literature (23, 26). The comprehensive assessment showed an increase in serum DAO levels compared to the control group (SMD = 2.51; 95% CI = 0.29–4.73; *p* = 0.03), with evidence of high heterogeneity (I^2^ = 98%) (Figure 6). The sequential exclusion of the included pieces of literature did not reduce heterogeneity.

## 4 Discussion

### 4.1 Main findings of the meta-analysis

The purpose of this systematic review is to summarize the evidence of IP in patients with CVDs. The combined results showed that the levels of IP markers in patients with CVDs were higher than those in the control group, indicating that CVD patients had characteristics of intestinal barrier damage. Except for zonulin, the heterogeneity of the combined analysis of the other markers is high and the results need to be carefully analyzed. Nevertheless, the markers of IP in patients with CVDs not only showed a single increase but also showed an increase in IP as the severity of the disease increased. Hu et al. (23) compared the IP of three groups of patients with coronary heart disease, and the results showed that the more severe the symptoms of the disease, the greater the IP. Niebauer et al. (19) found that the level of LPS in patients with chronic heart failure complicated with edema was higher than that in patients with stable chronic heart failure (0.74 EU/mL vs. 0.37 EU/mL). Therefore, patients with more severe CVDs should pay more attention to the condition of IP.

### 4.2 Strengths and weaknesses

The study was characterized by a comprehensive search, strict specification of inclusion and exclusion criteria, and more comprehensive outcome indicators. In addition, the study subjects included multiple countries, which reduces the variability due to ethnicity and better reflects the status of CVD in humans. However, experimental results can only indicate the status of IP in patients with CVDs but can not prove whether CVDs affect IP or whether the increased IP affects CVDs.

At present, most of the clinical studies on IP of CVDs are single-center and small-sample trials, and quite a few literature only use one outcome index to evaluate IP (20–22, 24–27), which has some limitations. The sugar probe test can evaluate IP by comparing the uptake of disaccharides and monosaccharides in the circulation. Because the collected sample is urine, it has limitations. In addition, the clearance rate of the liver and kidney seems to affect the test results. A clinical study showed that low urine recovery of sugar probes found in cirrhotic patients appears to be the result of nonintestinal factors affecting clearance rather than reduced intestinal absorption (32). Citrulline is a non-protein amino acid, and in humans, its plasma content is derived largely from the amount produced in enterocytes of the small bowel (33). The sensitivity and specificity of the citrulline test seem to be better than those of the sugar probe test (34). As a component of the cell wall of gram-negative bacteria, LPS can only be dissociated when the bacteria die and lyse. As an indicator of bacterial translocation, LPS cannot fully reflect the systemic and local inflammatory response. The detectable level in peripheral blood is low (35). There are three types of FABP (I-FABP, liver FABP, and leal bile acid binding protein), which are differentially expressed in different parts of the intestine. For example, I-FABP is mainly present in the jejunum (36). Other differentially expressed markers include serum DAO to evaluate the permeability of small intestine (37). I-FABP exists in the intestinal mucosa and d-lactate exists in the intestinal lumen, and the combination of the two has a certain complementarity. The above markers can reflect the local state of the intestine or IP, but each has its characteristics and limitations. The combined use of multiple markers is expected to become a reliable predictor of IP.

Except for the literature of Kim et al. (16), all participants included in the other literature were 48 years of age and older. The combined results may better reflect the IP of middle-aged and elderly patients with CVDs. Older patients should pay more attention to IP. There are divergences in the diagnostic criteria of CVDs in the included literature. For example, the type of disease studied by Sandek et al. (20) and Ahmad et al. (21) is chronic heart failure. Sandek et al. (20) took left ventricular ejection fraction ≤40% as one of the diagnostic criteria, while Ahmad et al. took left ventricular ejection fraction ≤50% as one of the diagnostic criteria. Moreover, the literature by Zhou et al. (28) did not describe a clear diagnostic criterion. The above factors have an impact on the quality of the study. In addition, in the included literature, the measurement methods of the same biomarker are different, and they are all cross-sectional studies, which may be the reason for the significant heterogeneity in outcomes. In summary, the conclusions of this study need to be further verified by increasing multi-center, large samples, multi-biomarker, and high-quality clinical randomized controlled trials to provide a more stable basis for the treatment of CVDs.

### 4.3 Research progress on the intervention of intestinal barrier and CVDs

CVDs are generally characterized by narrowing or occlusion of the blood supply of vascular beds (1, 2) resulting in insufficient blood perfusion of tissues and organs, which is one of the main factors causing intestinal ischemia (38), and then destroying the intestinal barrier and causing the increase of IP (39). Some literature has shown that increased IP can cause intestinal microbiota and metabolites (LPS, d-lactate, trimethylamine-N-oxide) to enter the blood circulation cause inflammation, and accelerate the development of CVDs (27, 40, 41). In addition, the effect of increased IP on intestinal microbiota also includes changes in quantity and loss of diversity (22, 42). Some intestinal microbiotas are directly related to CVDs and can even reflect the risk factors of CVDs. Kim et al. (16) used the Pearson correlation coefficient to analyze the correlation between systolic blood pressure and microbiota abundance (*p* < 0.05), results of eight kinds of microbiota with positive correlation with systolic blood pressure and three kinds of microbiota with negative correlation were identified. Intestinal microbiota has multiple effects, they can also directly affect IP, and pathogens can enhance the transfer of harmful substances in the blood and stimulate inflammatory responses (43); beneficial microbiota can maintain the integrity of IP and contribute to the reduction of IP. The relationship between CVDs, IP, and intestinal microbiota is not simple, but interactive.

Related basic and clinical studies have shown that the development of CVDs can be prevented by downregulating IP markers, regulating the intestinal microbiota, repairing tight junction proteins to restore the function of the intestinal barrier, and attenuating the inflammatory response (43, 44). Most CVDs require drug intervention, and these drugs also affect IP (45, 46). However, based on current literature, the effects of drugs for CVDs on IP are not the same. For example, hypertension drug captopril can down-regulate the level of IP markers and repair the function or structure of the intestinal barrier (46). Long-term use of atorvastatin will directly affect the intestinal microbiota and down-regulate the function of tight junction proteins to destroy the intestinal barrier (45). A study by Sandek et al. (20) found that IP in chronic heart failure patients taking low-dose aspirin was twice as high as in the control group. It can be seen that the drugs for the treatment of CVDs have different effects on IP, and the specific causes and mechanisms are still unclear. Cardiovascular drugs that hurt IP should be used with caution.

The intestinal microbiota is considered to be one of the key elements that help regulate host health. Members of the intestinal microbiota affect the metabolism and immune status of the host by regulating nutritional metabolism, drug metabolism, and the production of antibacterial metabolites, thus affecting the function of IP. Zhou et al. (28) used polymyxin B to treat myocardial infarction in mice. The mechanism of action is that it can inhibit intestinal microbiota translocation, thereby reducing the inflammatory response and inhibiting monocyte infiltration. Probiotics, as the god of intestinal protection, have become a hot topic in the field of research in recent years. While maintaining intestinal homeostasis, they also have a certain impact on the occurrence and development of CVDs. For example, in an *in vitro* study, Cheng et al. (45) used human colon carcinoma cell lines to verify that *Akkermansia muciniphila* can rescue intestinal barrier dysfunction caused by long-term use of atorvastatin. Another is fecal microbiota transplantation, whose potential for treating CVDs has been tested in experimental models. For example, Liu et al. (47) in a study revealed an important role in vascular dysfunction and metabolic disorder phenotypes by transplanting fecal microbiota from patients with coronary artery disease and healthy donors into germ-free mice. Meanwhile, the levels of ileal tight junction proteins such as claudin-1 and ZO-1 were significantly increased in mice transplanted with healthy donor fecal bacteria compared with those transplanted with coronary artery disease patients. The results indicated that mice transplanted with healthy donor fecal microbiota had enhanced intestinal barrier function and reduced IP. Therefore, it is very meaningful to improve host health by regulating intestinal microbiota and reducing IP.

Factors such as diet, exercise, alcohol, and age can also affect IP (48). Nutrients such as vitamins, amino acids, and dietary fiber maintain the homeostasis of different components of the intestinal mucosal barrier (49). For example, oat fiber reversed the increase in atherosclerotic lesions in LDLR−/− mice. It also increased the expression of tight junction proteins, including ZO-1 and occludin, and improved the intestinal mucosal barrier (50). In a cohort study, a single alcohol binge did not appear to alter intestinal barrier function (51). However, rats showed increased IP and intestinal oxidative damage after prolonged alcohol intake (52). Circadian rhythms are critical for maintaining the integrity of the intestinal barrier, and disruption of the biological clock promotes increased alcohol-induced IP (53). In addition, there is a link between aging and IP. In a clinical study, serum zonulin was found to be higher in older adults than in younger adults (*p* = 0.005). Zonulin was associated with muscle strength (r = −0.332, *p* = 0.048) and stamina (r = −0.410, *p* = 0.016) (54) In a 12-week exercise study, increased cardiorespiratory fitness led to relative improvements in markers of IP in patients with coronary artery disease (55). This suggests a potential mechanism by which prolonged exercise can improve gut barrier integrity. In short, the above factors provide more possibilities for the treatment and prevention of CVDs.

### 4.4 Summary and prospects

In total, 13 pieces of literature were integrated for meta-analysis in this systematic review, the results indicate that the IP of patients with CVDs increases, and IP markers may be used as one of the auxiliary diagnosis methods of CVDs. It is still not clear whether the change of IP is the result of the pathogenesis or pathogenic factors of CVDs, in the future, basic research should also strengthen the specific mechanism of action between CVDs and IP. In addition, some related studies do not evaluate IP or only measure a biomarker, which has some limitations, the evaluation criteria of IP should be improved in future research. Regulating IP may open up new avenues for the prevention and treatment of CVDs, but more attention should be paid to clinical research in this area in future research.

## Data availability statement

The original contributions presented in the study are included in the article/Supplementary material, further inquiries can be directed to the corresponding authors.

## Author contributions

J-HX: Conceptualization, Data curation, Methodology, Software, Writing – original draft, Writing – review & editing. YW: Conceptualization, Data curation, Writing – original draft. X-MZ: Conceptualization, Data curation, Writing – original draft. W-XW: Conceptualization, Data curation, Writing – original draft. QZ: Conceptualization, Data curation, Writing – original draft. Y-PT: Writing – review & editing. S-JY: Funding acquisition, Supervision, Writing – review & editing.
