# Supplementary material for: Intestinal permeability in human cardiovascular diseases: a systematic review and meta-analysis
Source: Front Nutr. 2024 Jul 17;11:1361126. doi: 10.3389/fnut.2024.1361126 (PMC11289889; doi:10.3389/fnut.2024.1361126)

Supplementary Material

# Supplementary Material 1

Supplementary Material 1 Database search strategy

1. PubMed

(((((((((((((((((((((((((((((((((((((((((((((cardiovascular diseases[MeSH Terms]) or (Cardiovascular Disease[Title/Abstract])) or (Disease, Cardiovascular[Title/Abstract])) or (Diseases, Cardiovascular[Title/Abstract])) or (hypertension[MeSH Terms])) or (Blood Pressure, High[Title/Abstract])) or (Blood Pressures, High[Title/Abstract])) or (High Blood Pressure[Title/Abstract])) or (High Blood Pressures[Title/Abstract])) or (coronary heart disease[MeSH Terms])) or (Coronary Diseases[Title/Abstract])) or (Disease, Coronary[Title/Abstract])) or (Diseases, Coronary[Title/Abstract])) or (Coronary Heart Disease[Title/Abstract])) or (Coronary Heart Diseases[Title/Abstract])) or (Disease, Coronary Heart[Title/Abstract])) or (Diseases, Coronary Heart[Title/Abstract])) or (Heart Disease, Coronary[Title/Abstract])) or (Heart Diseases, Coronary[Title/Abstract])) or (myocardial infarction[MeSH Terms])) or (Infarction, Myocardial[Title/Abstract])) or (Infarctions, Myocardial[Title/Abstract])) or (Myocardial Infarctions[Title/Abstract])) or (Cardiovascular Stroke[Title/Abstract])) or (Cardiovascular Strokes[Title/Abstract])) or (Stroke, Cardiovascular[Title/Abstract])) or (Strokes, Cardiovascular[Title/Abstract])) or (Myocardial Infarct[Title/Abstract])) or (Infarct, Myocardial[Title/Abstract])) or (Infarcts, Myocardial[Title/Abstract])) or (Myocardial Infarcts[Title/Abstract])) or (Heart Attack[Title/Abstract])) or (Heart Attacks[Title/Abstract])) or (atherosclerosis[MeSH Terms])) or (Atheroscleroses[Title/Abstract])) or (Atherogenesis[Title/Abstract])) or (Atherosclerotic[Title/Abstract])) or (Arrhythmias, Cardiac[MeSH Terms])) or (Arrhythmia, Cardiac[Title/Abstract])) or (Cardiac Dysrhythmia[Title/Abstract])) or (Dysrhythmia, Cardiac[Title/Abstract])) or (Cardiac Arrhythmia[Title/Abstract])) or (Cardiac Arrhythmias[Title/Abstract])) or (Arrhythmia[Title/Abstract])) or (Arrythmia[Title/Abstract]))

AND

((((((((((Lipopolysaccharides[Title/Abstract]) or (lipopolysaccharide-binding protein[Title/Abstract])) or (zonulin[Title/Abstract])) or (Citrulline[Title/Abstract]))) or (fatty acid binding proteins[Title/Abstract])) or (Lipopolysaccharide[Title/Abstract])) or (LPS[Title/Abstract]))

AND

((((((intestines[MeSH Terms]) or (gut[Title/Abstract])) or (gastrointestinal[Title/Abstract])) or (GI[Title/Abstract])) or (intestinal[Title/Abstract])) or (mucosal[Title/Abstract])))

AND

(((((((permeability[MeSH Terms]) or (permeability[Title/Abstract])) or (leaky[Title/Abstract])) or (hyperpermeability[Title/Abstract])) or (function[Title/Abstract])) or (dysfunction[Title/Abstract])) or (injury[Title/Abstract])))

2. Embase

No. Query Results

#1. (gut:ab,ti or gastrointestinal:ab,ti or gi:ab,ti or intestines:ab,ti or intestinal:ab,ti or mucosal:ab,ti)

AND

(permeability:ab,ti or leaky:ab,ti or hyperpermeability:ab,ti or function:ab,ti or dysfunction:ab,ti or injury:ab,ti)

AND

('cardiovascular diseases':ab,ti or hypertension:ab,ti or 'myocardial infarction':ab,ti or atherosclerosis:ab,ti or 'arrhythmias, cardiac':ab,ti or 'cardiovascular disease':ab,ti or 'disease, cardiovascular':ab,ti or 'diseases, cardiovascular':ab,ti or 'blood pressure, high':ab,ti or 'blood pressures, high':ab,ti or 'high blood pressure':ab,ti or 'high blood pressures':ab,ti or 'coronary diseases':ab,ti or 'disease, coronary':ab,ti or 'diseases, coronary':ab,ti or 'coronary heart disease':ab,ti or 'coronary heart diseases':ab,ti or 'disease, coronary heart':ab,ti or 'diseases, coronary heart':ab,ti or 'heart disease, coronary':ab,ti or 'heart diseases, coronary':ab,ti or 'infarction, myocardial':ab,ti or 'infarctions, myocardial':ab,ti or 'myocardial infarctions':ab,ti or 'cardiovascular stroke':ab,ti or 'cardiovascular strokes':ab,ti or 'stroke, cardiovascular':ab,ti or 'strokes, cardiovascular':ab,ti or 'myocardial infarct':ab,ti or 'infarct, myocardial':ab,ti or 'infarcts, myocardial':ab,ti or 'myocardial infarcts':ab,ti or 'heart attack':ab,ti or 'heart attacks':ab,ti or atheroscleroses:ab,ti or atherogenesis:ab,ti or atherosclerotic:ab,ti or 'arrhythmia, cardiac':ab,ti or 'cardiac dysrhythmia':ab,ti or 'dysrhythmia,cardiac':ab,ti or 'cardiac arrhythmia':ab,ti or 'cardiac arrhythmias':ab,ti or arrhythmia:ab,ti or arrythmia:ab,ti) AND (lipopolysaccharides:ab,ti or 'lipopolysaccharide-binding protein':ab,ti or zonulin:ab,ti or citrulline:ab,ti or 'fatty acid binding proteins':ab,ti or lipopolysaccharide:ab,ti or lps:ab,ti)

3. Cochrane library

#1 MeSH descriptor: [Intestines] explode all trees

#2 (gut or gastrointestinal or GI or intestinal or mucosal):ti,ab,kw

#3 MeSH descriptor: [Permeability] explode all trees

#4 (permeability or leaky or hyperpermeability or function or dysfunction or injury):ti,ab,kw

#5 #1 or #2

#6 #3 or #4

#7 #5 AND #6

#8 (Lipopolysaccharides or lipopolysaccharide-binding protein or zonulin or Citrulline or fatty acid binding proteins or Lipopolysaccharide or LPS):ti,ab,kw

#9 (#7 and #8)

#10 MeSH descriptor: [Atherosclerosis] explode all trees

#11 MeSH descriptor: [Cardiovascular Diseases] explode all trees

#12 MeSH descriptor: [Myocardial Infarction] explode all trees

#13 MeSH descriptor: [Hypertension] explode all trees

#14 MeSH descriptor: [Arrhythmias, Cardiac] explode all trees

#15 MeSH descriptor: [Coronary Disease] explode all trees

#16 (Cardiovascular Disease or Disease, Cardiovascular or Diseases, Cardiovascular or Blood Pressure, High or Blood Pressures, High or High Blood Pressure or High Blood Pressures or Coronary Diseases or Disease, Coronary or Diseases, Coronary or Coronary Heart Disease or Coronary Heart Diseases or Disease, Coronary Heart or Diseases, Coronary Heart or Heart Disease, Coronary or Heart Diseases, Coronary or Infarction, Myocardial or Infarctions, Myocardial or Myocardial Infarctions or Cardiovascular Stroke or Cardiovascular Strokes or Stroke, Cardiovascular or Strokes, Cardiovascular or Myocardial Infarct or Infarct, Myocardial or Infarcts, Myocardial or Myocardial Infarcts or Heart Attack or Heart Attacks or Atheroscleroses or Atherogenesis or Atherosclerotic or Arrhythmia, Cardiac or Cardiac Dysrhythmia or Dysrhythmia, Cardiac or Cardiac Arrhythmia or Cardiac Arrhythmias or Arrhythmia or Arrythmia):ti,ab,kw

#17 #10 or #11 or #12 or #13 or #14 or #15 or #16

#18 #9 AND #17

4. Web of Science

1: TS=(cardiovascular diseases or hypertension or coronary heart disease or myocardial infarction or atherosclerosis or Arrhythmias, Cardiac or Cardiovascular Disease or Disease, Cardiovascular or Diseases, Cardiovascular or Blood Pressure, High or Blood Pressures, High or High Blood Pressure or High Blood Pressures or CORonary Diseases or Disease, CORonary or Diseases, CORonary or CORonary Heart Disease or CORonary Heart Diseases or Disease, CORonary Heart or Diseases, CORonary Heart or Heart Disease, CORonary or Heart Diseases, CORonary or Infarction, Myocardial or Infarctions, Myocardial or Myocardial Infarctions or Cardiovascular Stroke or Cardiovascular Strokes or Stroke, Cardiovascular or Strokes, Cardiovascular or Myocardial Infarct or Infarct, Myocardial or Infarcts, Myocardial or Myocardial Infarcts or Heart Attack or Heart Attacks or Atheroscleroses or Atherogenesis or Atherosclerotic or Arrhythmia, Cardiac or Cardiac Dysrhythmia or Dysrhythmia, Cardiac or Cardiac Arrhythmia or Cardiac Arrhythmias or Arrhythmia or Arrythmia )

2: TS= (Lipopolysaccharides or lipopolysaccharide-binding protein or zonulin or Citrulline or fatty acid binding proteins or Lipopolysaccharide or LPS)

3: TS= (gut or gastrointestinal or GI or intestines or intestinal or mucosal)

4: TS= (permeability or permeability or leaky or hyperpermeability or function or dysfunction or injury)

5: #1 AND #2 AND #3 AND #4

5. ClinicalTrials.gov

(cardiovascular diseases or hypertension or myocardial infarction or atherosclerosis or arrhythmias, cardia) AND Intestinal permeability

6. Wanfang

主题:(肠道粘膜通透性 or 肠道屏障 or 肠道通透性 or 肠漏 or 肠粘膜屏障 or 肠道健康) and 主题:(心血管疾病 or 心脑血管疾病 or 心脑血管病 or 心血管病 or 心血管病变 or 心脑血管病变 or 动脉粥样硬化 or 动脉硬化 or 粥样动脉硬化 or 冠心病 or 冠状动脉粥样硬化心脏病 or 冠状动脉粥样硬化性 or 肺心痛 or 缺血性心脏病 or 肺心病人 or 冠状动脉硬化心脏病 or 慢性肺原性心脏病 or 缺血性心肌病 or 冠狀动脉性疾病 or 肺心病 or 冠状动脉心脏病 or 冠状动脉粥样硬化 or 冠状动脉粥样硬化症 or 心肌纤维化 or 真心痛 or 心肌梗死 or 急性心肌梗死 or 急性心肌硬死 or 心梗 or 急性心梗 or 急性心急梗死 or 心肌梗死 or 心肌梗塞 or 急性心机梗死 or 急性心肌梗死患者 or 急性心肌梗塞 or 老年急性心肌梗死 or 高血压 or 心律失常)

7. Weipu

题名或关键词=肠道粘膜通透性 or 肠道屏障 or 肠道通透性 or 肠漏 or 肠粘膜屏障 or 肠道健康

AND

题名或关键词=心血管疾病 or 心脑血管疾病 or 心脑血管病 or 心血管病 or 心血管病变 or 心脑血管病变 or 动脉粥样硬化 or 动脉硬化 or 粥样动脉硬化 or 冠心病 or 冠状动脉粥样硬化心脏病 or 冠状动脉粥样硬化性 or 肺心痛 or 缺血性心脏病 or 肺心病人 or 冠状动脉硬化心脏病 or 慢性肺原性心脏病 or 缺血性心肌病 or 冠狀动脉性疾病 or 肺心病 or 冠状动脉心脏病 or 冠状动脉粥样硬化 or 冠状动脉粥样硬化症 or 心肌纤维化 or 真心痛 or 心肌梗死 or 急性心肌梗死 or 急性心肌硬死 or 心梗 or 急性心梗 or 急性心急梗死 or 心肌梗死 or 心肌梗塞 or 急性心机梗死 or 急性心肌梗死患者 or 急性心肌梗塞 or 老年急性心肌梗死 or 高血压 or 心律失常

8. China National Knowledge Infrastructure

(主题:肠道屏障功能(精确)) OR (篇关摘:肠道粘膜通透性+肠道屏障+肠道通透性+肠漏+肠粘膜屏障+肠道健康(精确)) AND ((主题:心血管疾病(精确)) OR (篇关摘:心血管疾病+心脑血管疾病+心脑血管病+心血管病+心血管病变+心脑血管病变(精确)) OR (主题:动脉粥样硬化(精确)) OR (篇关摘:动脉粥样硬化+动脉硬化+粥样动脉硬化(精确)) OR (主题:冠心病(精确)) OR (篇关摘:冠心病+冠状动脉粥样硬化心脏病+冠状动脉粥样硬化性+肺心痛+缺血性心脏病+肺心病人+冠状动脉硬化心脏病+慢性肺原性心脏病+缺血性心肌病+冠狀动脉性疾病+肺心病+冠状动脉心脏病+冠状动脉粥样硬化+冠状动脉粥样硬化症+心肌纤维化+真心痛(精确)) OR (主题:心肌梗死(精确)) OR (篇关摘:心肌梗死+急性心肌梗死+急性心肌硬死+心梗+急性心梗+急性心急梗死+心肌梗死+心肌梗塞+急性心机梗死+急性心肌梗死患者+急性心肌梗塞+老年急性心肌梗死(精确)) OR (主题:高血压(精确)) OR (主题:心律失常(精确)))

9. China Biology Medicine disc

("肠道粘膜通透性"[常用字段] or "肠道屏障"[常用字段] or "肠道通透性"[常用字段] or "肠漏"[常用字段] or "肠粘膜屏障"[常用字段] or "肠道健康"[常用字段]) AND ("心血管疾病"[常用字段] or "心脑血管疾病"[常用字段] or "心脑血管病"[常用字段] or "心血管病"[常用字段] or "心血管病变"[常用字段] or "心脑血管病变"[常用字段] or "动脉粥样硬化"[常用字段] or "动脉硬化"[常用字段] or "粥样动脉硬化"[常用字段] or "冠心病"[常用字段] or "冠状动脉粥样硬化心脏病"[常用字段] or "冠状动脉粥样硬化性"[常用字段] or "肺心痛"[常用字段] or "缺血性心脏病"[常用字段] or "肺心病人"[常用字段] or "冠状动脉硬化心脏病"[常用字段] or "慢性肺原性心脏病"[常用字段] or "缺血性心肌病"[常用字段] or "冠狀动脉性疾病"[常用字段] or "肺心病"[常用字段] or "冠状动脉心脏病"[常用字段] or "冠状动脉粥样硬化"[常用字段] or "冠状动脉粥样硬化症"[常用字段] or "心肌纤维化"[常用字段] or "真心痛"[常用字段] or "心肌梗死"[常用字段] or "急性心肌梗死"[常用字段] or "急性心肌硬死"[常用字段] or "心梗"[常用字段] or "急性心梗"[常用字段] or "急性心急梗死"[常用字段] or "心肌梗死"[常用字段] or "心肌梗塞"[常用字段] or "急性心机梗死"[常用字段] or "急性心肌梗死患者"[常用字段] or "急性心肌梗塞"[常用字段] or "老年急性心肌梗死"[常用字段] or "高血压"[常用字段] or "心律失常"[常用字段])

# Supplementary Table S1 Summary of characteristics of the included studies and populations studied.

| **Study** | **Mean age (years)** | **Gender** | **Types of disease** | **Methods of diagnosis** | **Outcome measures** |
| --- | --- | --- | --- | --- | --- |
| Hu 2022 [13] | 50 | 56% M  44% F | CHD | Coronary CT angiography and coronary angiography | ①②③ |
| You 2019 [14] | 67 | 57% M  43% F | CHD + AMI | Coronary angiography: at least one major coronary artery with a diameter stenosis of ≥ 50% to form the CHD group, and those with angiographic evidence of intracoronary thrombosis diagnosed as AMI were the AMI group. | ① |
| Kim 2018 [15] | ＞18 | Not reported | HTN | HBP was defined as SBP ≥ 140 mmHg, the prehypertensive cohort as SBP from 120 to 139 mmHg and the reference cohort as SBP ≤ 130 mmHg. | ①④⑤ |
| Li 2016 [16] | 63 | 49% M  51% F | CAD | The diagnostic criterion for CAD was the patient having more than 50% luminal diameter narrowing of the vessel. | ④ |
| Loffredo 2022 [17] | 62 | 43% M  57% F | MVA | (1) Presence of symptoms of myocardial ischemia; (2) Objective documentation of myocardial ischemia, as assessed by currently available techniques; (3) Absence of obstructive CAD ( <50% coronary diameter reduction and/or fractional flow reserve > 0.80) documented by CT coronary scan or coronary angiography; (4) Confirmation of a reduced coronary blood flow reserve and/or inducible microvascular spasm. | ①④ |
| Niebauer 1999 [18] | 61 | Not reported | CHF | Based on symptoms arising during exercise, cardiomegaly, and documented left-ventricular dysfunction (all patients had a left-ventricular ejection fraction measured by echocardiography or radionuclide ventriculography of < 40%). | ①⑥ |
| Sandek 2012 [19] | 68 | Not reported | CHF | Based on a history of typical symptoms of at least 6 months, cardiomegaly, and documented left ventricular dysfunction (left ventricular ejection fraction ≤ 40%). | ①⑦ |
| Zhou 2018 [20] | 60 | 75% M  25% F | CHD + STEMI | Not reported | ①② |
| Ahmad 2022 [21] | Control：54-73  Patient：56-74 | 62%M  38% F | CHF | Left ventricular ejection fraction < 50%, including HF patients with reduced ejection fraction and patients with mildly reduced ejection fraction. | ④ |
| Yang 2016 [22] | 53.86 | 62% M  38% F | ATAAD | Based on typical clinical signs, echocardiography, and enhanced CT of the coarcted aneurysm. | ③ |
| Liu 2020 [23] | 48 | 70% M  30% F | NCHD + CHD | The patients were divided into two groups according to the results of coronary angiography: coronary artery stenosis > 50% was the CHD group, and coronary artery stenosis < 50% was the NCHD group. The patients in the NCHD group had coronary risk factors, including HTN, dyslipidemia, arrhythmia, and ischemic changes evident on an electrocardiogram. | ① |
| Cheng 2023 [24] | 51 | 71% M  29% F | CHD | Patients were divided into two groups based on the results of their coronary angiography. Patients with >50% coronary artery stenosis were assigned to the CHD group, and patients with < 50% coronary artery stenosis were assigned to the NCHD group. | ① |
| Carnevale 2018 [25] | 72 | 76% M  24% F | Atherosclerotic | Critical stenosis of the carotid artery > 70% were diagnosed with atherosclerotic disease. | 1. ④ |

Abbreviations: AMI, acute myocardial infarction; HTN, hypertension; HBP, high blood pressure; SBP, systolic blood pressure; CAD, coronary artery disease; MVA, microvascular angina; CT, computed tomography; CHF, chronic heart failure; STEMI, ST-segment elevation myocardial infarction; HF, heart failure; ATAAD, acute type A aortic dissection; NCHD, non-stenosis coronary heart disease

Note：① LPS; ② d-lactate; ③ serum DAO; ④ zonulin; ⑤ I-FABP; ⑥ LBP; ⑦ melibiose/rhamnose

# Supplementary Table S2 The quality assessments of each included study.

| Study | Q.1 | Q.2 | Q.3 | Q.4 | Q.5 | Q.6 | Q.7 | Q.8 | Risk of Bias |
| --- | --- | --- | --- | --- | --- | --- | --- | --- | --- |
| Hu 2022 | Yes | Yes | Yes | Yes | Yes | uncertain | Yes | Yes | Low Risk |
| You 2019 | Yes | Yes | Yes | Yes | uncertain | uncertain | Yes | Yes | Low Risk |
| Kim 2018 | Yes | uncertain | Yes | Yes | No | uncertain | Yes | Yes | Moderate Risk |
| Li 2016 | Yes | Yes | Yes | Yes | No | No | Yes | Yes | Low Risk |
| Loffredo 2022 | Yes | Yes | Yes | Yes | Yes | uncertain | Yes | Yes | Low Risk |
| Niebauer 1999 | Yes | Yes | Yes | Yes | No | No | Yes | Yes | Low Risk |
| Sandek 2012 | Yes | No | Yes | uncertain | Yes | uncertain | Yes | Yes | Moderate Risk |
| Zhou 2018 | Yes | Yes | Yes | uncertain | Yes | uncertain | Yes | Yes | Low Risk |
| Ahmad 2022 | Yes | Yes | Yes | Yes | uncertain | uncertain | Yes | Yes | Low Risk |
| Yang 2016 | Yes | Yes | Yes | Yes | Yes | uncertain | Yes | Yes | Low Risk |
| Liu 2020 | Yes | Yes | Yes | uncertain | Yes | Yes | Yes | Yes | Low Risk |
| Cheng 2023 | Yes | Yes | uncertain | uncertain | Yes | Yes | uncertain | Yes | Moderate Risk |
| Carnevale 2018 | No | No | Yes | uncertain | Yes | No | Yes | Yes | Moderate Risk |

Note: Q.1: Were the criteria for inclusion in the sample clearly defined? Q.2: Were the study subjects and the setting described in detail? Q.3: Was the exposure measured in a valid and reliable way? Q.4: Were objective standard criteria used for measurement of the condition? Q.5: Were confounding factors identified? Q.6: Were strategies to deal with confounding factors stated? Q.7: Were the outcomes measured in a valid and reliable way? Q.8: Was appropriate statistical analysis used?

# Supplementary Figure 1 The Funnel plot analysis of the studies included in the analysis.


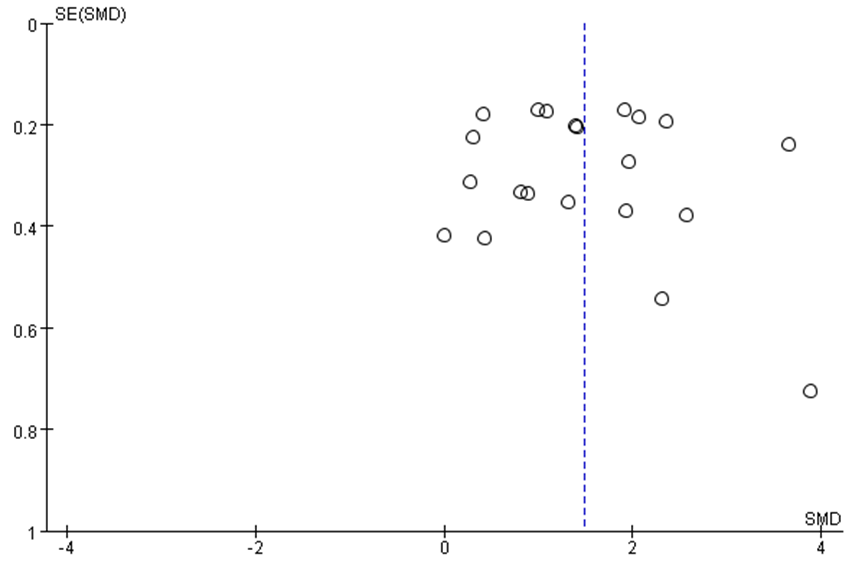


# The Egger test of the studies included in the analysis.


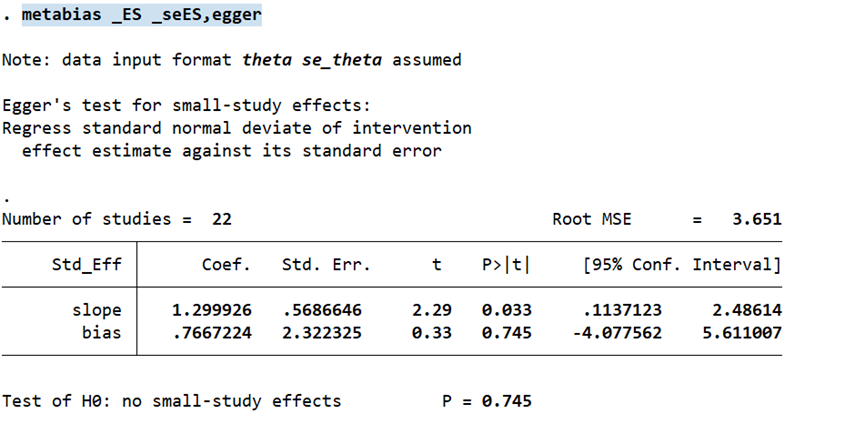

Supplement: Supplementary file 1 [file Data_Sheet_1.DOCX]
